# Identification of novel sepsis-related neutrophil subsets and surface markers for clinical diagnosis and outcome

**DOI:** 10.1080/21505594.2026.2723631

**Published:** 2026-08-25

**Authors:** Simiao Chen, Xuanwen Ru, Ting Zhang, Danlei Chen, Qingyi Shao, Ruiying Liu, Hao Zhou, Wenxia Shao, Qing Ye

**Affiliations:** a Department of Clinical Laboratory, Children’s Hospital, Zhejiang University School of Medicine, Hangzhou, China; b School of Medical Technology and Information Engineering, Zhejiang Chinese Medical University, Hangzhou, China; c Department of Laboratory Medicine, Affiliated Hangzhou First People’s Hospital, School of Medicine, Westlake University, Hangzhou, China

**Keywords:** Sepsis, neutrophils, diagnosis, CD54, CD62L

## Abstract

Sepsis, a life-threatening immune dysregulation caused by infection, lacks specific laboratory indicators for differential diagnosis and outcome assessment. This study aimed to identify immune cell heterogeneity and surface characteristic molecules for sepsis diagnosis and outcome evaluation. Transcriptomic and proteomic profiling were designed to identify immune cell-related molecules and form a detection panel for cytometry by time-of-flight (CyTOF) analysis. CyTOF revealed three neutrophil subsets: TGF-β^hi^CD62L^low^ (immunosuppressive phenotype), CD54^+^CD62L^+^ (proinflammatory phenotype), and CD54^low^CD62L^+^ (compensatory immunosuppressive phenotype), correlating with sepsis severity, progression and prognosis. In the acute stage of sepsis, the number of CD54^+^ and CD62L^+^ neutrophils increased, and their expression gradually decreased as the course of the disease progresses; however, their expression in the mild group decreased rapidly and then stabilized, whereas that in the severe group decreased slowly. At 24 h, the severe group retained higher CD54^+^ (*p* = 0.0051) and CD62L^+^ (*p* = 0.0003) expression. Serum sCD54 in sepsis patients (PXD027485 dataset) was elevated vs controls (*p* < 0.0001, AUC = 0.9627). Parallel reaction monitoring (PRM) analysis confirmed higher sCD54 in sepsis cohorts (*p* = 0.0242, AUC = 0.9433). Plasma exosomal CD54 in septic rats also increased (*p* = 0.0013). In conclusion, three identified neutrophil subsets and CD54 serve as sepsis-specific indicators for outcome assessment and differential diagnosis, guiding personalized therapies.

## GRAPHICAL ABSTRACT

**Figure:**
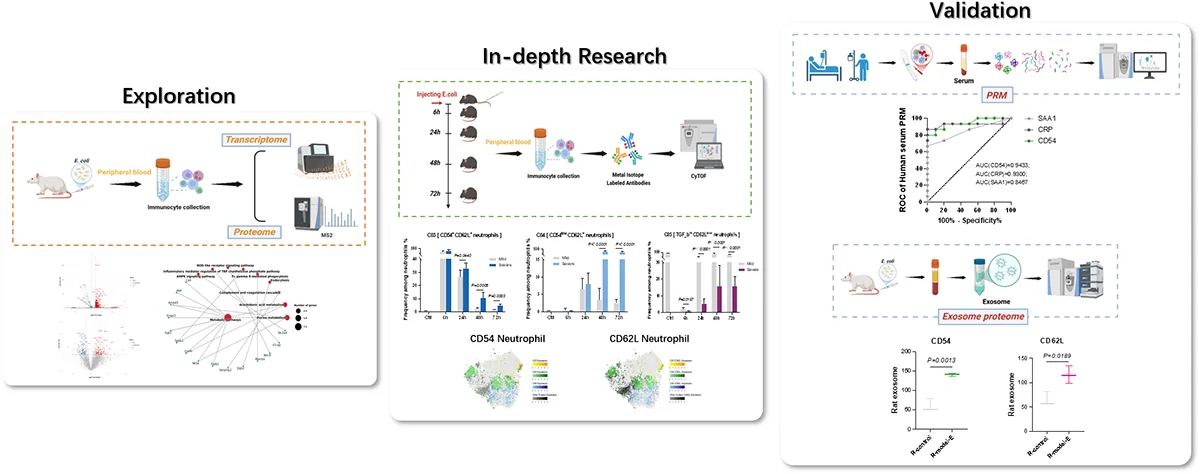

## Introduction

The essence of sepsis, a systemic inflammatory response triggered by infection, is that microorganisms or their products invade the blood circulation and activate the immune system, triggering a series of complex physiological responses. According to the latest Sepsis-3 definition, this disease originates from the dysregulation of the host response to infection, which leads to life-threatening organ dysfunction, which is still a major challenge in the global critical care field [1]. Currently, the diagnosis of sepsis mainly depends on a series of clinical criteria, in which the sequential [Sepsis-related] Organ Failure Assessment (SOFA) is particularly critical. These criteria accurately determine sepsis cases by identifying patients with two or more sepsis features combined with evidence of infection [2]. However, the existing diagnostic methods have significant limitations [3]. On the one hand, the early symptoms of sepsis are nonspecific and are easily confused with those of other diseases, which makes early diagnosis difficult [4,5]. On the other hand, although blood culture, CRP, PCT level examinations and other detection methods can aid in diagnosis [6], blood culture is time-consuming and prone to false-negative results, while CRP and PCT, as acute-phase reactants, often lack sufficient sensitivity and specificity in the early course of the disease [7]. In addition, some patients may not exhibit typical sepsis features due to a low microbial load or atypical antigen presentation mechanisms [8]. Therefore, the development of novel laboratory indicators with high specificity for early differential diagnosis and prognostic evaluation of sepsis has become a critical scientific challenge in clinical practice.

As a core part of the immune system, immune cells play a critical role in maintaining the body’s immune defense. In acute inflammatory states such as sepsis, changes in immune cells, such as increased white blood cell counts and decreased lymphocyte proportions, are a concrete reflection of the body’s emergency response to infection or injury [9]. As the main force of immune cells, neutrophils play a pivotal role in the rapid response in the early stage of infection [10]. They are attracted by chemokines to the site of infection and defend against infection through phagocytosis and bactericidal effects [11]. Studies have shown that neutrophils exhibit significant heterogeneity in various diseases, and different subgroups exhibit unique functions and behaviors in inflammation, autoimmune diseases, the tumor microenvironment, etc [10]. In sepsis, highly active neutrophils can release large amounts of oxygen free radicals and enzymes to induce an inflammatory storm, thus aggravating tissue damage [12]. In chronic inflammatory and autoimmune diseases, abnormally activated neutrophils may lead to persistent inflammation and tissue destruction [13]. In addition, specific subgroups of neutrophils play important roles in the regulation of the immune response and the inhibition of T-cell function [14,15]. These studies elucidated the complex roles of neutrophils and their subgroups in the maintenance of immune balance and the occurrence and development of disease and provided a solid theoretical basis for the development of targeted treatment strategies.

This study aimed to integrate transcriptomic and proteomic data to construct a high-dimensional immune analysis platform. Through the use of CyTOF and high-dimensional data analysis technology, the heterogeneity and surface characteristic molecules of neutrophil subsets are analyzed in depth, and clinical sample validation is carried out. This study provides a scientific basis for the differential diagnosis and disease outcome assessment of sepsis.

## Materials and methods

### Ethics approval and consent to participate

This study adhered to the Declaration of Helsinki and ARRIVE guidelines. All animal procedures were approved by the Animal Care and Use Committee of Zhejiang Eyong Pharmaceutical Research and Development Center (Approval No: ZJEY-20230420-04). And the clinical retrospective data analysis study was approved by the Ethics Committee of the Children’s Hospital, Zhejiang University School of Medicine (Approval No: 2023-IRB-0107-P-01). The committee also waived the requirement for informed consent, as this study involved only the retrospective analysis of patient medical records and the examination of remaining clinical serum samples, no additional serum samples were collected. The data were anonymized and accessible only to the research team.

### Rat experimental design

Twenty-two adult male SD rats weighing 250–280 g (Slack Laboratory Animal Co., Ltd., Hangzhou, CN) were randomly divided into two groups: the control group (no treatment) and the sepsis group (sepsis modeling as per a previous study [16]), with 11 rats in each group. After 72 h of modeling, peripheral blood samples were collected via the abdominal aorta under continuous inhalation anesthesia with isoflurane (RWD Life Science Co., Ltd., Shenzhen, CN) administered via a small animal anesthesia machine. Among them, eight samples from each group were used for transcriptome analysis. Another three samples were selected for proteome analysis.

### Mouse experimental design

A total of 272 C57BL/6 male mice (Qizhen Laboratory Animal Co., Ltd., CN) with body weights ranging from 24–26 g were selected and randomly divided into a control group (24 mice), a mild sepsis group (118 mice), and a severe sepsis group (130 mice). The sepsis model was established according to the methods of a previous study, and the injection volume was adjusted according to the experimental needs: each animal in the mild group was injected with 2 × 10^7^ CFU, and those in the severe group were injected with 1 × 10^8^ CFU per animal. Ten animals in the mild group and the severe group were randomly selected for 7-day survival analysis, respectively. After modeling, at 6, 24, 48, and 72 h, peripheral blood was collected from the surviving mice through the orbital vein for CyTOF.

### Serum and data collection from sepsis patients

Serum samples remaining from routine clinical tests were collected from 15 patients meeting the Sepsis 3.0 diagnostic criteria and 10 healthy controls. Relevant data were retrospectively gathered. General information collected included age, gender, and the following laboratory parameters: Mean arterial pressure, White blood cell count, Hemoglobin, Hematocrit, Platelet count, Albumin, Aspartate aminotransferase, Alanine aminotransferase, Total bilirubin, Creatinine, INR. Additionally, the source of infection, vasopressor use, and mechanical ventilation status were recorded.

### Transcriptome analysis of rat peripheral blood immune cells

First, RNA from immune cells was isolated and purified from the peripheral blood of the rats. Quality control was performed via a NanoDrop ND-1000 (NanoDrop, Wilmington, DE, USA) and a Bioanalyzer 2100 (Agilent, CA, USA) to ensure that the concentration, purity, and integrity of the RNA met the standards (concentration > 50 ng/μL, RIN value > 7.0, and amount of total RNA > 1 μg). The mRNA was subsequently captured and fragmented by oligo (dT) magnetic beads (Dynabeads Oligo (dT), Thermo Fisher, USA) and a magnesium ion fragmentation kit (NEBNextR Magnesium RNA Fragmentation Module, USA), followed by reverse transcription to synthesize cDNA. The composite duplex was converted to a DNA duplex by *E. coli* DNA polymerase I and RNase H, and dUTP was incorporated in the process to fill the end and add an A base. After digestion with the UDG enzyme, PCR amplification was performed to form a strand-specific library with a length of 300 bp ±50 bp. Finally, paired-end PE150 sequencing was performed via an Illumina NovaSeqTM 6000 (LC Bio Technology Co., Ltd. Hangzhou, CN).

### Proteome analysis of rat peripheral blood immune cells and plasma exosomes

In this study, mass spectrometry and DDA were performed on immune cells and plasma exosomal peptides in rat peripheral blood via the Vanquish Neo UHPLC system and an Orbitrap Exploris 480 mass spectrometer (Thermo Scientific™, San Jose, USA). The specific procedure was as follows: peptides first entered the precolumn at a flow rate of 6 μL/min and then entered the analytical column at a flow rate of 300 nL/min for 60-min gradient analysis. The scan parameters of the mass spectrometer included FAIMS voltages of −45 V and −65 V, a cycle time of 1 second, a primary scan range of 375–1,800 m/z, a resolution of up to 60,000, an AGC target value of 300%, a maximum ion injection time of 50 ms, and a low signal intensity. The ions at 2e4 were not collected. The secondary resolution was set to 30,000, the AGC target value was 200%, the maximum ion injection time was 86 ms, TurboTMT was set to TMTpro Reagent, the selective isolation window of the precursor ion was 0.7 m/z, and the scan initiation mass was 110 m/z.

### CyTOF analysis of mouse peripheral blood immune cells

Immune cells from the peripheral blood of the mice were extracted, stained for metal-labeled surface markers and fixed. After the samples were mixed, they were detected at a rate of 300 events/s via a Helios mass spectrometer (Standard BioTools, USA). After signal correction, decoding, and manual gating preprocessing, the original data were used for cluster analysis via the PhenoGraph algorithm in R 4.3.3. All the effective cells in all the samples were clustered, and all the markers were selected. After arcsinh transformation, the data were visually analyzed via various methods, such as t-distributed stochastic neighbor embedding (t-SNE) maps, heatmaps, and histograms, to visualize conclusions with biological significance.

### PRM analysis of clinical peripheral blood sera

Serum samples from septic patients and healthy people who met the Sepsis 3.0 criteria were separated. After protein extraction and high-abundance protein removal, peptides were prepared. The peptides were separated via ultrahigh-performance liquid chromatography (UPLC), and the peptides were ionized by a capillary ion source before being analyzed via a timsTOF Pro mass spectrometer. The mass spectrometer was used in prm-PASEF mode. The charge number of the precursor ions was set to 0–5, and the scan range of the secondary mass spectrometer was 100–1700 m/z. The mass spectrometry data were retrieved via MaxQuant, and the database used was Homo_sapiens_9606_SP_20230103.fasta. Trypsin/P was selected for the enzyme digestion method, the number of missed cleavage sites was set to 2, the minimum length of the peptides was set to 7 amino acid residues, and the maximum number of modifications was set to 5. The mass error tolerance of both the primary precursor ions and the secondary fragment ions was set to 20 ppm. The fixed modifications are cysteine alkylation and carbamidomethyl (C), whereas the variable modifications include the oxidation of methionine and the acetylation of the N-terminus of the protein. The FDRs of protein identification and PSM identification were both set to 5%. Skyline 21.2 software was used for data processing. Trypsin [KR/P] was selected as the protease, the maximum number of missed cleavage sites was set to 0, the length of the peptides was set to 7–25 amino acid residues, and the fixed modification was cysteine alkylation. The transition parameters were set to 2 and 3 for the parent ion charge, 1 for the product ion charge, and b and y for the ion type. We chose to start matching from the third fragment ion, and the mass error tolerance was set to 0.02 Da.

### Data analysis

Blinding procedures were implemented during both the experimental treatments and data analysis processes. Descriptive statistics for numerical data were calculated using the median and interquartile range (25th to 75th percentile). The correlation analysis between the clinical datasets used the Spearman rank correlation coefficient (r) and the corresponding test methods, and a linear regression line was drawn on the chart to visualize the correlation. Routine variance analysis was performed via SPSS 25.0 software and GraphPad Prism 8, and the data were presented as the means ± SDs. All the data were statistically evaluated via a *t* test and a Mann‒Whitney *U* test, and differences with *a p* value less than 0.05 were considered statistically significant.

## Results

### Integrated transcriptome and proteome analysis of circulating immune cells in sepsis rats and exploration of differential molecular biological functions

**Figure 1.**
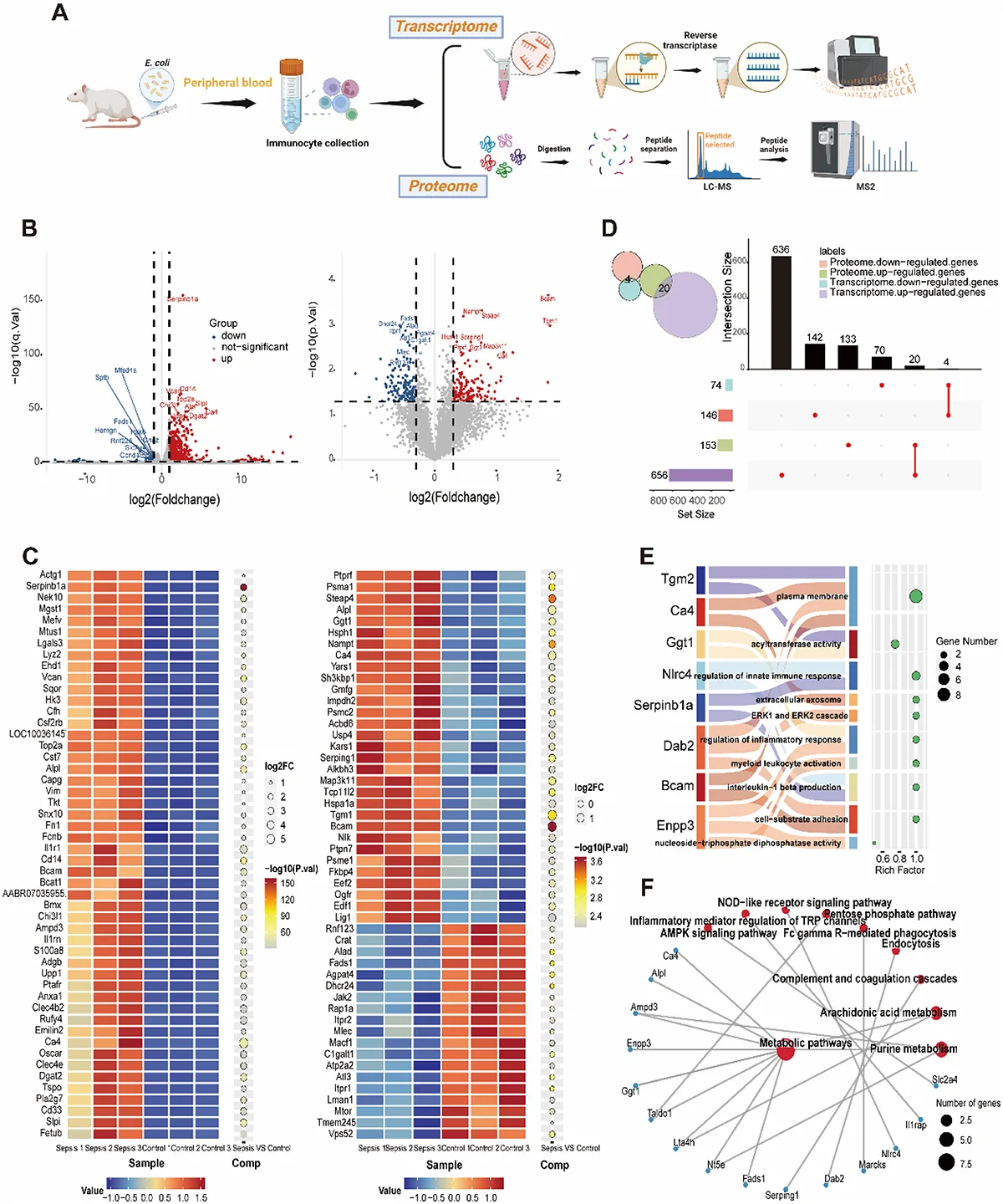
Transcriptome and proteome analysis of circulating immune cells in the peripheral blood of septic rats. (A) detection flow chart: this figure outlines the overall process of transcriptome and proteome analysis. (B) volcano plot showing the DEGs/proteins in the transcriptome (left) and proteome (right), with the top 10 upregulated and downregulated molecules, respectively. (C) presentation of the heatmap and bubble map: the transcriptome is shown on the left, and the proteome is shown on the right, both showing the relative levels of the top 50 DEGs/DEPs, which are visually represented by the depth of color and the size of the bubbles. (D) upset-venn diagram analysis: the numbers of upregulated and downregulated genes/proteins in the transcriptome and proteome, as well as the intersection between them, are shown. (E) Sankey bubble plot revealing the enrichment of the intersection genes/proteins in GO. The size of the bubbles and the thickness of the connecting lines indicate the degree of enrichment and relationship. (F) the network diagram shows the enrichment of the intersection genes/proteins in the KEGG pathway. The nodes represent genes/proteins, and the edges represent the interactions and pathway relationships between them.

In this study, circulating immune cells were isolated from the peripheral blood of septic rats, and the transcriptome and proteome were systematically detected Figure 1(A). Transcriptome analysis revealed 20,157 genes, whereas proteome analysis revealed 3546 proteins. Compared with the results of the control group, the transcriptome analysis revealed 730 differentially expressed genes (DEGs) (q < 0.05, log2FC > 1), with the expression of 656 genes upregulated and the expression of 74 genes downregulated. Moreover, proteome analysis revealed 299 differentially expressed proteins (DEPs) (*p* < 0.05, log2FC > 0.3), including 153 upregulated proteins and 146 downregulated proteins Figure 1(B). To visually display these significantly differential molecules, a bubble heatmap was used to present the relative expression levels of the top 50 differential genes/proteins. The results revealed that the expression of significantly different genes in the transcriptome was mainly upregulated, while both upregulated and downregulated proteins accounted for a certain proportion of the proteome Figure 1.

Through joint analysis of the transcriptome and proteome, this study identified 24 expressed molecules whose expression significantly differed at the two omics levels, with the expression of 20 molecules upregulated and the expression of 4 molecules downregulated Figure 1. To further explore the biological functions of these 24 differentially expressed molecules, enrichment analysis via Gene Ontology (GO) and Kyoto Encyclopedia of Genes and Genomes (KEGG) analyses was performed. The results of the GO analysis revealed that these differential molecules were located mainly in the plasma membrane and exosomes and participated in key biological processes, such as the regulation of innate immunity, cell adhesion, myeloid leukocyte activation, and the regulation of inflammation Figure 1(E). In addition, KEGG pathway enrichment analysis revealed that 15 differential molecules were enriched in 10 key signaling pathways, including purine metabolism and arachidonic acid metabolism linked to chemotaxis, AMPK and TRP signaling pathways associated with cell migration, NOD-like receptors, FcγR-mediated phagocytosis, etc Figure 1(F).

### CyTOF-based phenotypic profiling and immune cell subpopulation characterization in the peripheral blood of sepsis mice

**Figure 2.**
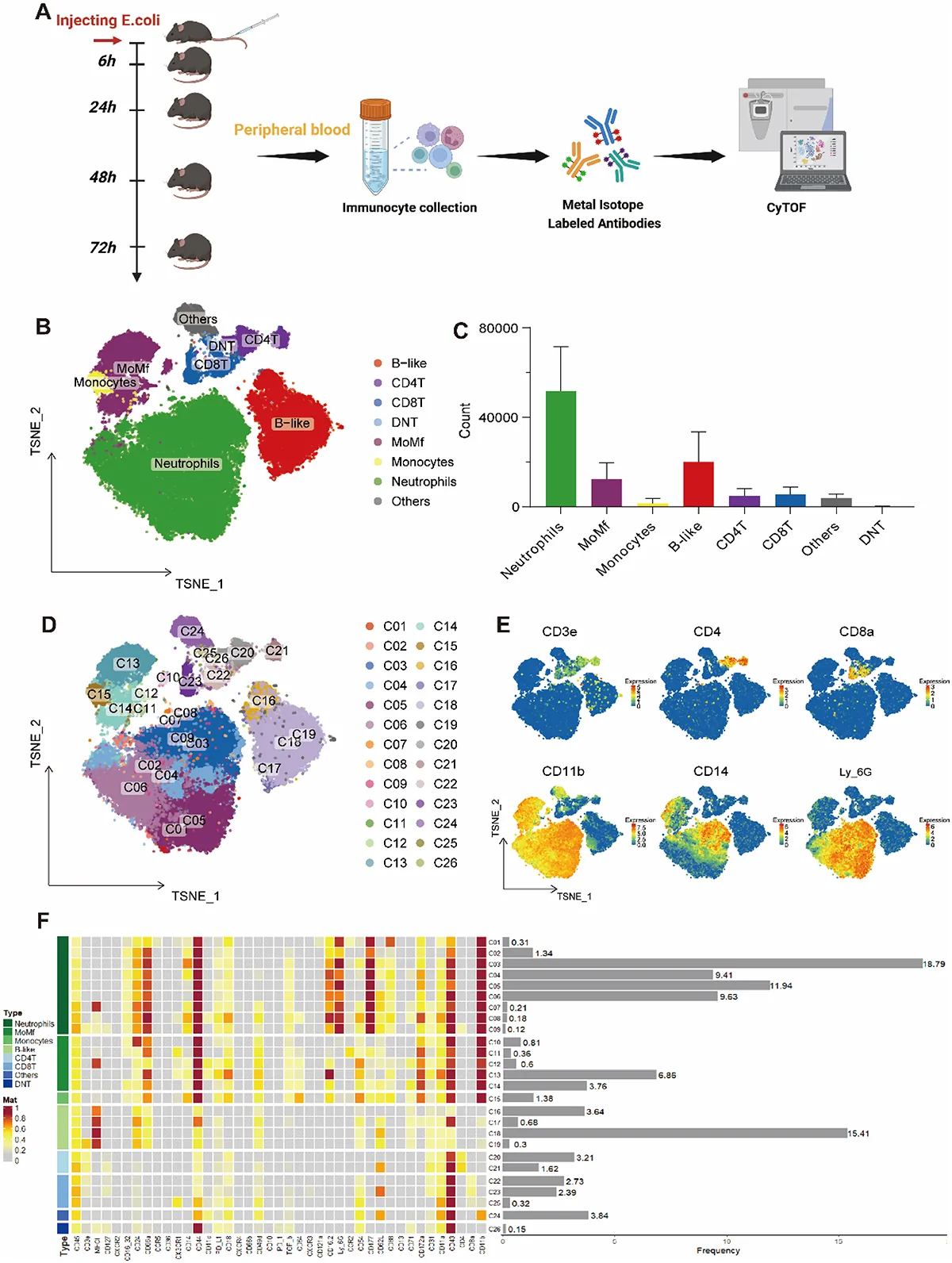
Overview of the phenotype atlas of circulating immune cells in the peripheral blood of septic mice. (A) flowchart of CyTOF. (B) t-SNE map of immune cell types in the whole sample. (C) histogram of the number of immune cells of each type in the whole sample. (D) t-SNE map of immune cell clusters in the whole sample. (E) t-SNE map of landmark marker expression levels in the whole sample. (F) heatmap of unsupervised clustering algorithm analysis of cell surface markers of different cell types and clusters.

On the basis of the aforementioned results of joint omics analysis, the present study revealed that sepsis-specific molecules were involved mainly in core mechanisms such as chemotaxis, migration, and phagocytosis of immune cells. Accordingly, a set of tailored panels (Supplementary Table S1) was designed, and a comprehensive and in-depth assessment of circulating immune cells was performed via CyTOF Figure 2(A). On the basis of the expression patterns of the markers, this study successfully identified eight major immune cell types Figure 2(B). In terms of the number distribution, neutrophils accounted for the greatest number in the peripheral blood, followed by B cells Figure 2. In addition, the study also clustered and annotated 26 cell subgroups, of which the C01-C09 subgroups were all neutrophil subgroups Figure 2. Moreover, this study highlighted the expression levels of representative markers in the overall maps, including CD3e (T-cell marker), CD4 (T helper T-cell marker), CD8a (cytotoxic T-cell marker), CD11b (monocyte and macrophage marker), CD14 (monocyte marker), and Ly_6G (neutrophil marker) Figure 2(E). In addition, the results of the phenotype clustering heatmap visually revealed that among the circulating immune cell population, the number of neutrophils accounted for the majority, and among neutrophils, the C03‒C06 subgroups were the main component Figure 2(F).

### Dynamic changes and functional enrichment analysis of neutrophil subsets in sepsis mice

**Figure 3.**
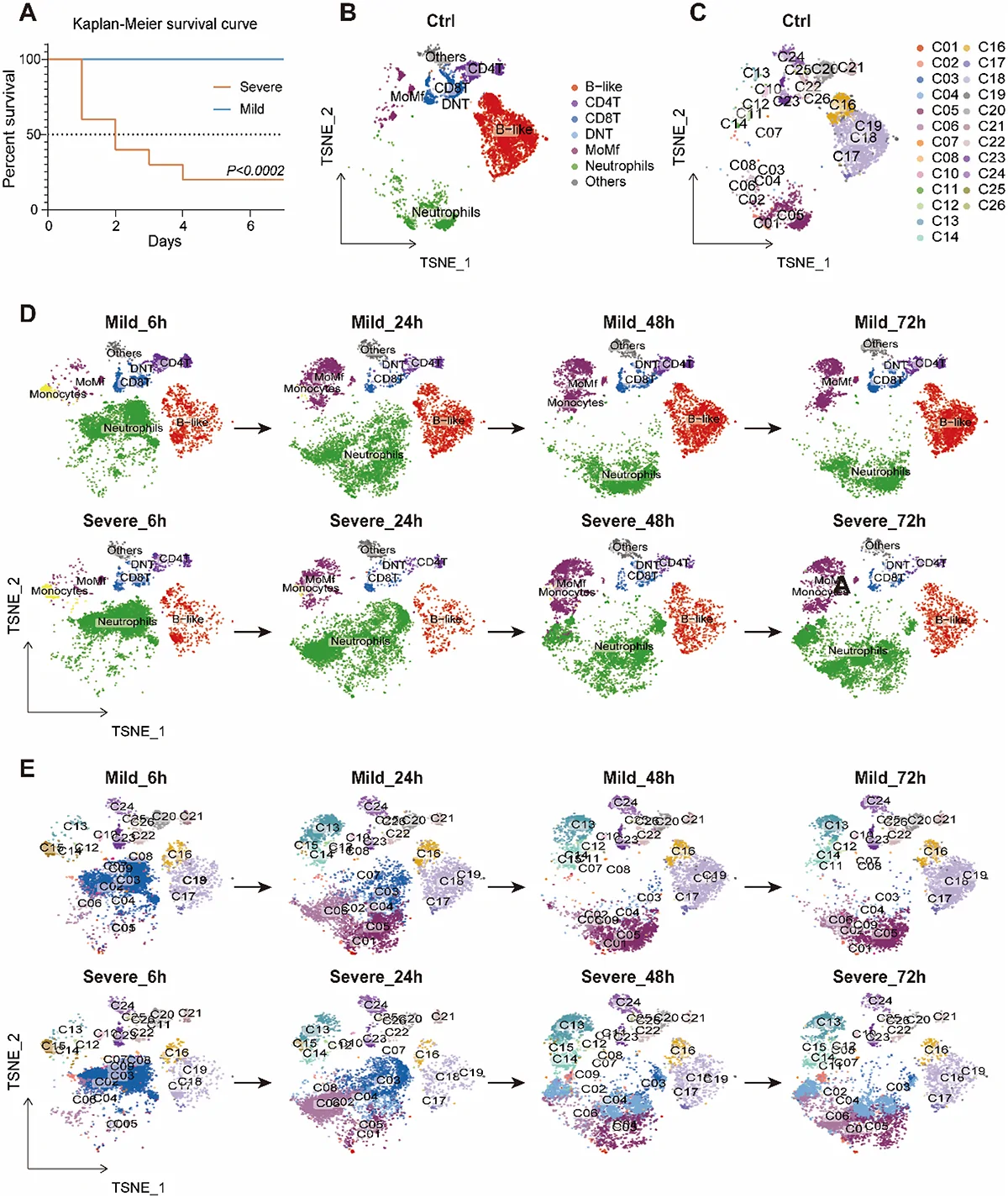
Survival curves of mice with different disease outcomes and t-SNE maps of peripheral blood immune cells. (A) Kaplan‒Meier survival curves of mice with different severities of sepsis. (B and C) t-SNE maps of circulating immune cell types and clusters in the mice in the control group. (D and E) t-SNE maps of circulating immune cell types and clusters in septic mice as a function of the course and severity of the disease.

**Figure 4.**
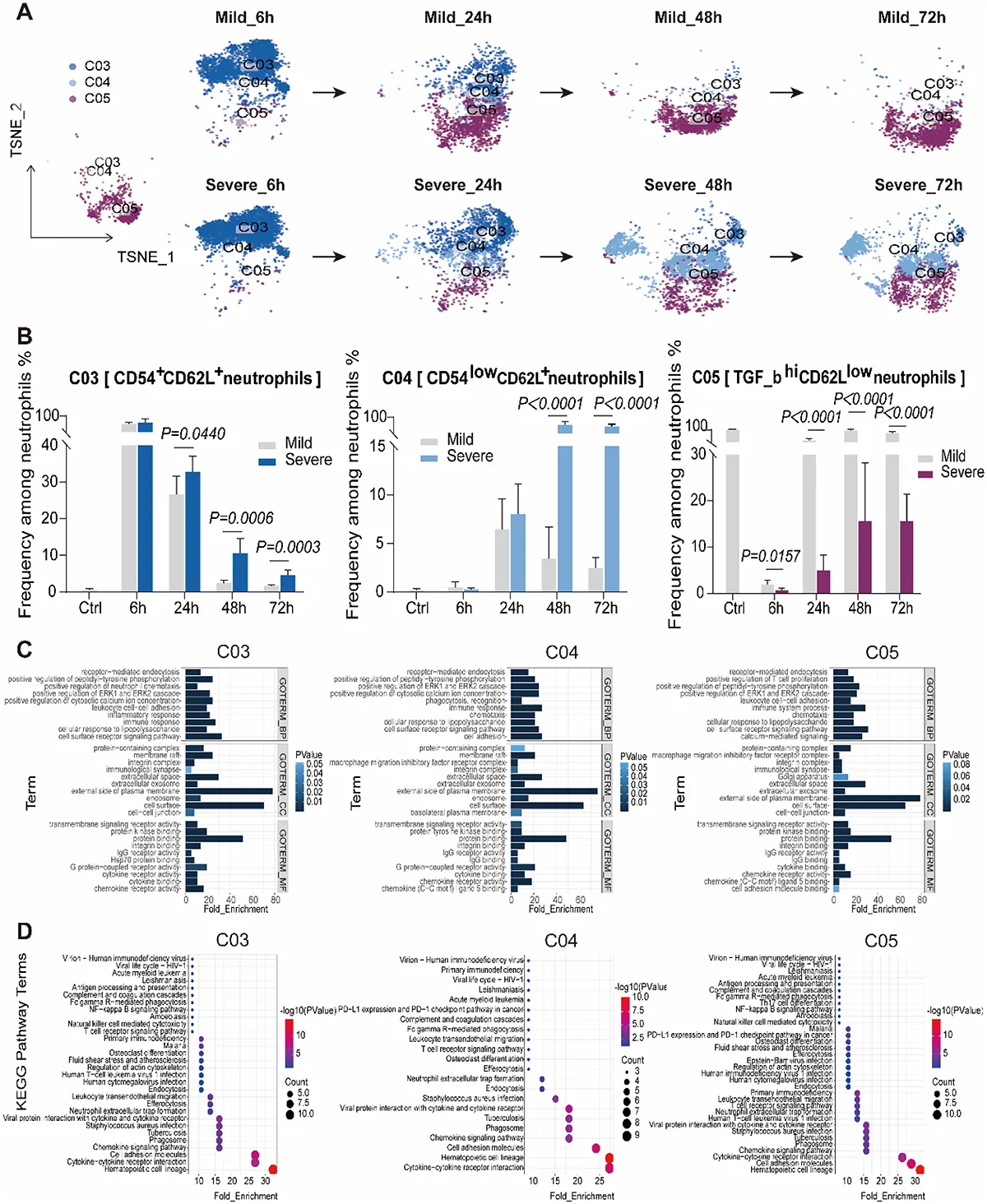
Disease outcome-associated dynamics of neutrophil subsets and functional enrichment profiles. (A) t-SNE maps of circulating neutrophils in septic mice as a function of disease course and severity. (B) histogram of the changes in the numbers of three specific neutrophil subsets with different courses and severities. (C and D) GO enrichment and KEGG pathway enrichment of three specific neutrophil subsets surface markers.

This study established sepsis mouse models covering different stages of the disease course (early, advanced, convalescent, and end-stage) and different degrees of severity (mild, severe). Survival curve analysis revealed that the survival rate of the mice in the mild group was 100%, whereas that of the severe group was only 20% Figure 3(A). In healthy control mice, relatively few neutrophils were detected Figure 3(B,C). However, in septic mice, neutrophil counts were markedly elevated, with a progressive increase in circulating neutrophils at 6 h and 24 h during disease progression. As the course of the disease progressed, the number of neutrophils in the mice in the mild group gradually decreased, whereas that in the severe group remained high Figure 3. Further analysis of the circulating neutrophil subsets revealed that three neutrophil subsets, C03 (CD54^+^CD62L^+^), C04 (CD54^low^CD62L^+^) and C05 (TGF-β^hi^CD62L^low^), presented the most significant changes Figure 3(E). Individual analysis of these three key subgroups via t-SNE maps, combined with their proportional changes in neutrophils, revealed that the TGF-β^hi^CD62L^low^ neutrophil subset was dominant in healthy mice, whereas the CD54^+^CD62L^+^ and CD54^low^CD62L^+^ neutrophil subsets were rare. In the acute stage (6 h) of sepsis, the CD54^+^CD62L^+^ neutrophil subset increased significantly, whereas the TGF-β^hi^CD62L^low^ neutrophil subset sharply decreased, and the CD54^low^CD62L^+^ neutrophil subset remained minimal. After 6 h, the CD54^+^CD62L^+^ neutrophil subset declined gradually, with a slower reduction in the severe group, with significant differences at 24 h (*p* = 0.0440), 48 h (*p* = 0.0006) and 72 h (*p* = 0.0003). The CD54^low^CD62L^+^ neutrophil subset increased markedly at 24 h, with subsequent changes linked to disease severity: it decreased in the mild group but increased continuously in the severe group, with significant differences at 48 h (*p* < 0.0001) and 72 h (*p* < 0.0001). The TGF-β^hi^CD62L^low^ neutrophil subset recovered gradually after 6 h, but recovery was slower in the severe group, with significant differences at 6 h (*p* = 0.0157), 24 h (*p* < 0.0001), 48 h (*p* < 0.0001) and 72 h (*p* < 0.0001) Figure 4(A-B). Furthermore, compared with the parameter panel, all the markers positively expressed on the surface of the three subsets were subjected to GO and KEGG enrichment analyses. The results revealed that the heterogeneity of these three neutrophil subsets was closely related to neutrophil chemotaxis, NET formation, FcγR-mediated phagocytosis, chemokine signaling pathway activity, cell adhesion, and leukocyte transendothelial migration Figure 4(C-D).

### Dynamic changes in CD54^+^ and CD62L^+^ neutrophil expression during sepsis progression

**Figure 5.**
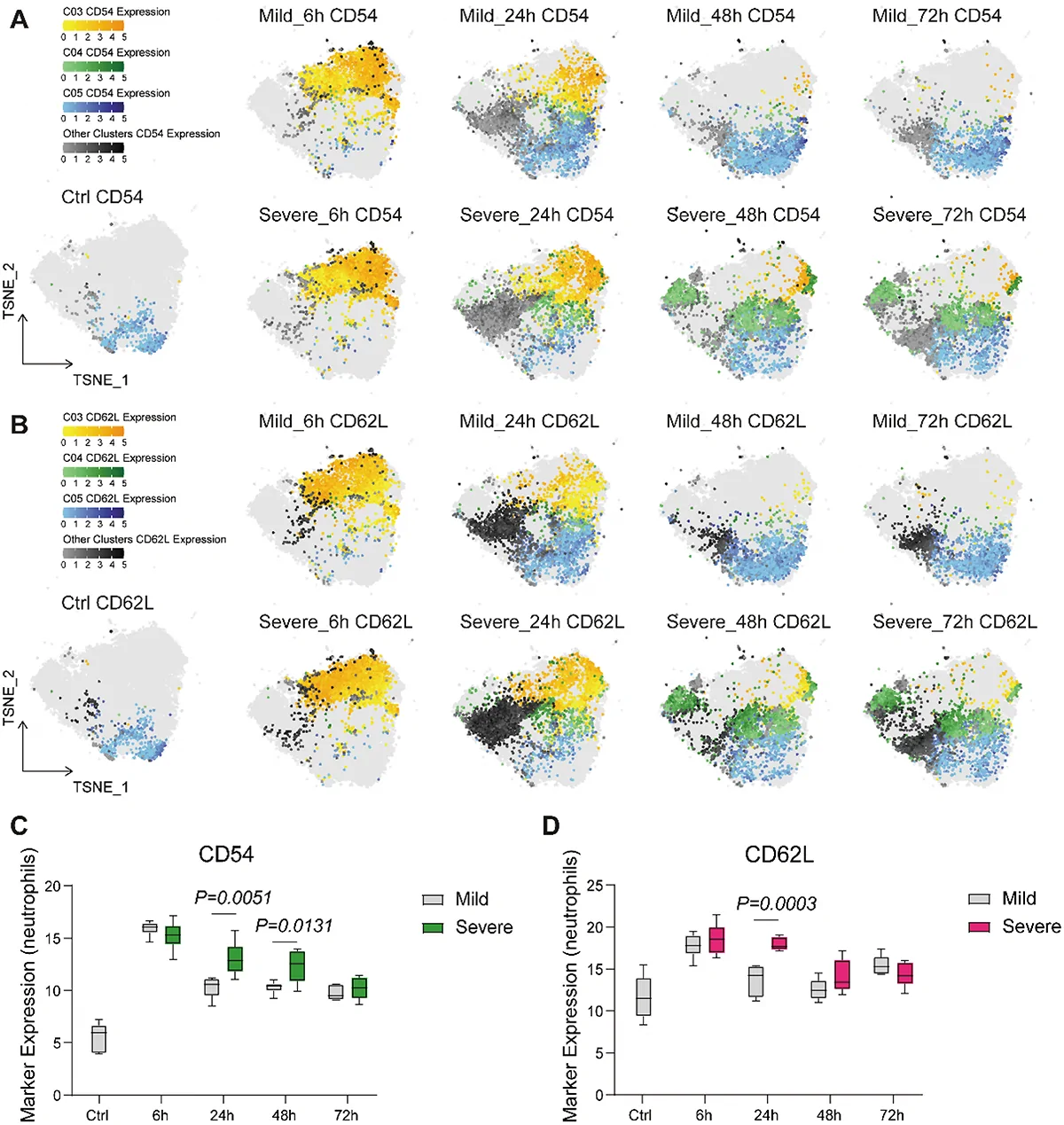
Expression of CD54 and CD62L in circulating neutrophils. (A and B) t-SNE maps of CD54^+^ neutrophils and CD62L^+^ neutrophils as a function of disease course and severity; surface CD54 and CD62L expression levels in C03 (CD54^+^CD62L^+^), C04 (CD54^low^CD62L^+^), C05 (TGF-β^hi^CD62L^low^), and other neutrophil subpopulations are visually represented by yellow, green, blue, and black gradient color scales, respectively. Neutrophils from nonmatching groups are shown in gray. (C and D) the expression levels of CD54 and CD62L in neutrophils with different disease courses and severities.

A comprehensive analysis of the above three unique neutrophil subsets revealed that the characteristic molecules on the surface were CD54 and CD62L. To further investigate the change patterns of these molecules in the ensemble of neutrophils, CD54 and CD62L were included in the ensemble-level analysis. The t-SNE dimensionality reduction algorithm was used to visualize the expression levels of CD54 and CD62L through the color gradient Figure 5(A-B). The findings further intuitively revealed that the subpopulations that effectively reflected sepsis progression and severity under the CD54 and CD62L expression patterns were still predominantly the CD54^+^CD62L^+^, CD54^low^CD62L^+^, and TGF-β^hi^CD62L^low^ neutrophil subsets, with the remaining neutrophil subpopulations showing irregular dynamic changes. During the acute phase of sepsis (6 h), the total CD54^+^ and CD62L^+^ neutrophil counts and expression levels increased in both the mild and severe groups and were concentrated in the CD54^+^CD62L^+^ subset. As the disease progressed to 24 h, the total CD54^+^ and CD62L^+^ neutrophil numbers continued to rise, with a slight decrease in CD54^+^CD62L^+^ as well as marked increases in the CD54^low^CD62L^+^ and TGF-β^hi^CD62L^low^ subsets. In contrast, overall CD54 and CD62L expression levels declined after 6 h, with a gradual decrease in the severe group and stabilization after an initial decrease in the mild group. At 24 h, the CD54 (*p* = 0.0051) and CD62L (*p* = 0.0003) levels remained significantly greater in the severe group. Notably, differences in CD54 expression persisted at 48 h (*p* = 0.0131), with higher total CD54^+^ neutrophil and CD54^low^CD62L^+^ subset counts in the severe group. Eventually, as the CD54 and CD62L levels in both groups decreased toward control levels, the numerical disparity persisted Figure 5(C-D).

### Soluble CD54 (sCD54) and CD62L (sCD62L) for the differential diagnosis of sepsis and analysis of expression pathways

**Figure 6.**
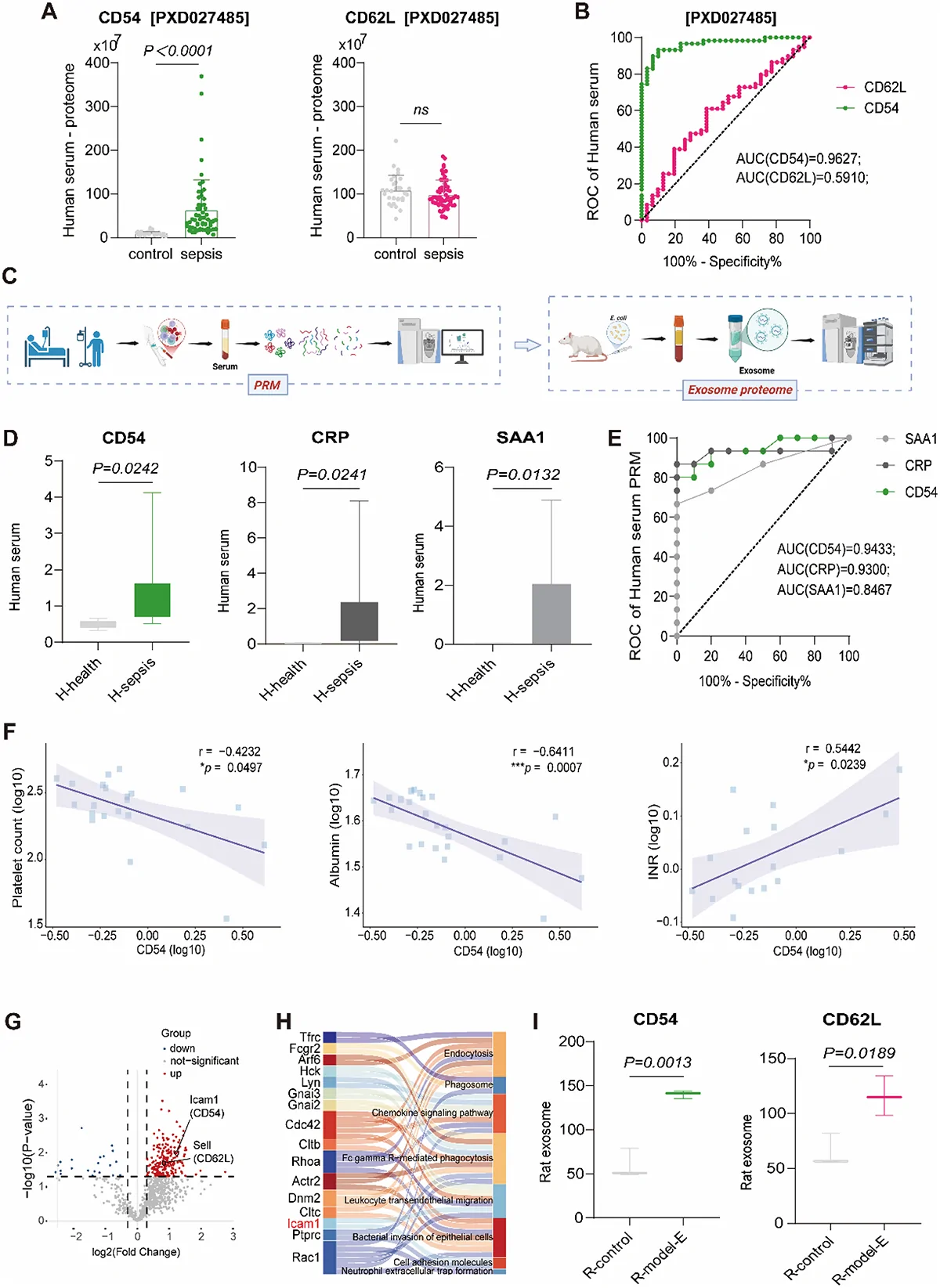
The expression of sCD54 and sCD62L. (A and B) the expression of sCD54 and sCD62L in the serum of septic patients was determined on the basis of the publicly available dataset PXD027485 and their ROC curves. (C) flowchart of clinical PRM detection and proteomic detection of exosomes in rat peripheral blood plasma. (D and E) the results of clinical PRM and the ROC curves of sCD54, CRP, and SAA1. (F) Spearman correlation analysis of sCD54 expression and the platelet count, albumin level, and INR in patients with clinical sepsis. (G) volcano plot showing the DEPs from the rat exosome proteome, in which CD54 and CD62L were directly annotated. (H) Sankey diagram showing the KEGG pathway enrichment of the DEPs in the rat exosomes. (I) the expression levels of CD54 and CD62L in rat exosomes.

Considering the convenience of clinical testing and the availability of samples, this study further focused on the quantitative analysis of sCD54 and sCD62Lin serum. The results of the proteome analysis based on the publicly available dataset PXD027485 revealed that the sCD54 level in the serum of septic patients was significantly greater than that in healthy controls (*p* < 0.0001), whereas the sCD62L level was not significantly different Figure 6(A). The diagnostic performance was evaluated via a receiver operating characteristic (ROC) curve, and the area under the curve (AUC) of sCD54 reached 0.9627 Figure 6(B).

To verify the above findings, this study collected serum samples from septic patients and healthy individuals (Supplementary Table S2 for the clinical characteristics). The PRM technique was used to quantitatively detect sCD54, the routine laboratory detection indicator C-reactive protein (CRP), and serum amyloid A1 (SAA1) Figure 6. Compared with healthy controls, sepsis patients presented significantly increased levels of sCD54 (*p* = 0.0242), CRP (*p* = 0.0241), and SAA1 (*p* = 0.0132) Figure 6. The AUC of sCD54 (0.9433) was greater than those of CRP (0.9300) and SAA1 (0.8467) Figure 6(E). In addition, Spearman correlation analysis further revealed that sCD54 was significantly correlated with the clinical platelet count (*r* = −0.6232; *p* = 0.0497), albumin level (*r* = −0.6411; *p* = 0.0007), and international normalized ratio (INR) (*r* = −0.5442; *p* = 0.0007) Figure 6(F).

To further elucidate the expression pathways of CD54 and CD62L in vivo and the communication patterns mediated between subgroups, this study conducted proteome analysis on plasma exosomes from a rat sepsis model Figure 6. A total of 898 proteins were identified, among which 237 were differentially expressed (*p* value < 0.05, fold change > 1.2, FDR < 1%), including 216 upregulated proteins and 21 downregulated proteins. Notably, both CD54 and CD62L were significantly upregulated in exosomes Figure 6(G). KEGG enrichment analysis of these DEPs revealed that CD62L-related pathways were not significantly enriched, whereas other representative pathways included chemokine signaling pathways, leukocyte transendothelial migration, and NET formation. Among these pathways, the pathways affected by CD54 were associated primarily with cell migration and adhesion Figure 6(H). Further quantitative analysis revealed that the levels of CD54 (*p* = 0.0013) and CD62L (*p* = 0.0189) in rat plasma exosomes were significantly greater than those in the control group Figure 6.

## Discussion

Sepsis is a complex disease of the systemic inflammatory response [17]. The functional heterogeneity of neutrophils plays a key role in their pathogenesis. Given that neutrophils have a short half-life [18], research should focus on their dynamic phenotypic changes in the blood circulation [19]. In recent years, the rapid development of omics technologies and large-scale cell analysis technologies has provided a new perspective for in-depth analysis of the immune characteristics of sepsis [20,21]. This study used integrated transcriptomics and proteomics methods to identify differentially expressed molecules closely associated with sepsis. These molecules are involved in functions such as chemotaxis and the migration of immune cells. On the basis of these results, a targeted detection parameter panel was designed and used in CyTOF. Changes in neutrophil subsets are closely associated with the course and severity of sepsis. Specifically, the TGF-β^hi^CD62L^low^, CD54^+^CD62L^+^ and CD54^low^CD62L^+^ neutrophil subsets could effectively reflect the severity, progression and prognosis of sepsis, respectively. To investigate in depth the change patterns of these molecules in the whole body of neutrophils, this study focused on the global analysis of CD54 and CD62L in neutrophils. The results showed that in the acute stage of sepsis, both the number and the expression of CD54^+^ and CD62L^+^ neutrophils increased, which provides an important basis for stratifying the diagnosis and treatment of this disease. Considering the convenience of clinical testing and the availability of samples, the sCD54 and sCD62L levels in the serum were further analyzed. The results revealed that sCD54 was closely associated with coagulation dysfunction and endothelial injury in sepsis and that the exosome-mediated secretion pathway may be the key mechanism by which CD54 regulates the immune response, whereas the secretion of CD62L may be more dependent on the classical pathway.

The functional diversity of neutrophils in sepsis has received widespread attention, but the subset-specific regulatory mechanisms are still not fully understood. This study revealed that CD54^+^CD62L^+^ neutrophils significantly expand in the early stage of sepsis, which may be closely related to the proinflammatory phenotype of this subset. CD54 (ICAM-1) promotes the migration of neutrophils to sites of inflammation by mediating the adhesion of leukocytes to vascular endothelial cells [22], and CD62L (L-selectin) provides a time window for subsequent effects through the rolling effect in the initial adhesion stage [23]. This finding is highly consistent with the classic three-step model of leukocyte migration (rolling-activation-solid adhesion) [24]; however, its dynamic attenuation in sepsis suggests the aggravation of the immunosuppressive state in the later stage. Notably, the CD54^+^CD62L^+^ neutrophil subset exhibited a faster recovery ability in the mild group, whereas the recovery process of this cell subgroup in the severe group was relatively slow, which reflects the differences in the ability to rebuild immune homeostasis. Studies have shown that the chemotaxis and bactericidal functions of neutrophils in severe sepsis are impaired by a reduced mitochondrial membrane potential and reduced ATP production [25]. In mild patients, the Treg cell-mediated IL-10 and TGF-β signaling pathways may promote the transformation of neutrophils to the anti-inflammatory phenotype, thereby accelerating the reconstruction of immune homeostasis [26,27]. In addition, the level of autophagy in neutrophils is increased in mild patients, which helps remove damaged mitochondria and maintains cellular functional homeostasis [28,29]. In critically ill patients, metabolic disorders lead to autophagy defects, which lead to excessive inflammation and tissue damage [30].

The dynamic changes in the CD54^low^CD62L^+^ neutrophil subset revealed another aspect of the functional plasticity of neutrophils. The persistent increase in this subset in the severe group may reflect a compensatory immunosuppressive phenotype [31]. A study by Pillay et al. [32] showed that neutrophils with low CD62L expression can inhibit T-cell proliferation through the release of arginase-1, thereby forming a local immunosuppressive microenvironment. However, the decreasing trend of this subset in the mild group suggests that its function is a double-edged sword: moderate immunosuppression may help limit inflammatory damage, whereas excessive accumulation exacerbates immune paralysis [33]. This paradoxical phenomenon may be related to the level of reactive oxygen species (ROS) in the microenvironment. The overproduction of ROS in severe sepsis may modify the CD62L domain through oxidative modification and hinder its binding with ligands [34–36]. As a result, the dysfunctional CD54^low^ CD62L^+^ neutrophil subset is retained in the circulation.

The dynamic changes in the TGF-β^hi^CD62L^low^ neutrophil subset further highlighted the dual roles of neutrophils in immune regulation. As a potent immunosuppressive factor, TGF-β can reduce the secretion of proinflammatory cytokines through the inhibition of related pathways [27,37]. This study revealed that the level of the TGF-β^hi^CD62L ^low^ neutrophil subset, which is increased in healthy mice, was significantly reduced in the acute stage of sepsis, suggesting that it may maintain immune homeostasis by suppressing excessive inflammation. However, the delayed recovery in the critically ill group may be related to persistent inhibition of the TGF-β signaling pathway in the microenvironment. This finding aligns with the observations reported by Sun et al. [38] in the sepsis model, although the specific regulatory mechanisms still need further exploration.

From the perspective of translational medicine, this study further focused on CD54 and CD62L, revealing their significant potential as candidate biomarkers of sepsis. The AUC value of sCD54 demonstrated statistically significant superiority over the conventional biomarkers CRP and SAA1, suggesting its enhanced diagnostic potential for early-stage sepsis detection. Moreover, the strong correlation between sCD54 and coagulation disorders (such as thrombocytopenia and increased international normalized ratio (INR)) further expands its pathological significance. Some studies have shown that sCD54 may exacerbate microvascular thrombus formation by promoting platelet‒leukocyte aggregation [39]. This mechanism involves the synergy of integrin αMβ2 (Mac-1) and P-selectin on the surface of platelets to activate the coagulation cascade and destroy endothelial barrier function [40], eventually leading to multiorgan microcirculation disorders. In addition, the high expression of CD54 in exosomes in the rat model was consistent with its serum level, suggesting that exosomes may be the main source of sCD54. The dynamic distribution and trafficking of CD54 are strictly regulated by inflammatory signals, and its presence in exosomes further expands its biological functions.

In contrast, the clinical application value of CD62L is controversial. Although the significant upregulation of CD62L in exosomes in the rat model suggested that it may be involved in inflammation amplification through vesicle trafficking, the stability of sCD62L in human serum may be due to the species-specific preference of the secretory pathway. Notably, the localization specificity of CD62L in lymph node high endothelial venules (HEVs) [41] showed that its biological significance may be reflected more in local tissues than in the circulatory system. Therefore, it is difficult to capture the whole picture of the dynamic regulation of CD62L in a single peripheral blood test. The sampling of multiple sites (such as lymphatic tissue and inflammatory foci) should be combined for comprehensive assessment. Future studies can explore the correlation between sCD62L and local immune cell infiltration to improve its reliability as a stratification marker of sepsis.

In addition, molecules such as CD177, CD11b, and CXCR2 are key functional molecules that regulate neutrophil activation, adhesion, migration, and chemotaxis [42–44]. Existing studies indicate that these molecules exhibit characteristic changes in expression during inflammation and sepsis, making them important biomarkers reflecting the functional status of neutrophils [45–47]. However, this study aims to focus on the association between neutrophil subpopulation characteristics and the course and severity of sepsis. Based on previous exploratory findings, CD177 expression showed no significant correlation with disease severity, CD11b lacked specificity, and CXCR2 expression levels were too low to meet clinical translation needs. Therefore, these molecules were not selected as core analytical targets for this study. Nevertheless, the aforementioned molecules retain significant biological relevance in the regulation of neutrophil function. Concurrently, neutrophil functional exhaustion has emerged as a new research paradigm in the field of sepsis, with pathogenic inflammatory dysregulation and innate immune dysfunction jointly driving disease progression [48]. Compared to changes in cell numbers, neutrophil functional exhaustion and pathological phenotypic reprogramming are considered the core mechanisms determining the severity of sepsis and its prognosis [49], and they represent an important direction worthy of in-depth exploration and systematic analysis in future research on the immunological mechanisms of sepsis.

Although we successfully identified three neutrophil subsets closely linked to sepsis outcomes, the resolution limitations of conventional flow cytometry hindered their precise isolation and functional validation, impeding further mechanistic exploration. To address this, we redirected our focus to their surface markers (CD54/CD62L) and serum-detectable surrogate molecules, aiming to promote clinical translation. Additionally, although the number of peripheral blood samples from mice used for proteomic analysis is not large, subsequent validation using clinical cohorts and public datasets further confirmed the reliability of our findings. The findings of this study provide a clear roadmap for future investigations to dissect the precise molecular mechanisms of these TGF-β^hi^CD62L^low^, CD54^+^CD62L^+^ and CD54^low^CD62L neutrophil subsets. For example, conditional gene knockout or adoptive transfer experiments, combined with animal models of sepsis, could be used to assess the causal effects of these subsets on bacterial clearance, inflammation regulation, and prognosis.

In summary, immune dysfunction in sepsis is a multidimensional and dynamically evolving process. CD54 was validated as a novel serum biomarker, while the dynamic expression of both CD54 and CD62L on neutrophil subsets provides critical insights for disease monitoring and outcome assessment. Simultaneously, the heterogeneity of neutrophil subsets and exosome-mediated signaling interactions between subsets offer new directions for immunological and metabolic interventions in sepsis. Future studies may necessitate enhanced integration of mechanistic investigations with translational implementation to overcome prevailing diagnostic–therapeutic limitations, thereby advancing clinical prognostication in sepsis care.

## Consent for publication

All authors have read and approved the submission of the manuscript.

## Supplementary Material

Supplementary.docx

## Data Availability

The data from this study is included in the main text/supplementary documents. For further inquiries, please contact the corresponding author. The raw data and R code have been uploaded to the public repository Zenodo and can be accessed via the following link: https://doi.org/10.5281/zenodo.20285390 [50]. The omics data can be accessed through the following link: Serum proteomic data of septic patients: https://www.iprox.cn//page/SCV017.html?query=PXD027485 Transcriptome data of peripheral blood immune cells in septic rats: https://www.ncbi.nlm.nih.gov/sra/?term=PRJNA1121656 Proteome data of peripheral blood immune cells in septic rats: https://proteomecentral.proteomexchange.org/cgi/GetDataset?ID=PXD053096 Proteome data of plasma exosomes from septic rats: http://proteomecentral.proteomexchange.org/cgi/GetDataset?ID=PXD072277
