# Supplementary material for: Identification of novel sepsis-related neutrophil subsets and surface markers for clinical diagnosis and outcome
Source: Virulence. 2026 Aug 25;17(1):2723631. doi: 10.1080/21505594.2026.2723631 (PMC13523946; doi:10.1080/21505594.2026.2723631)
Supplement: Supplementary file 1 [file KVIR_A_2723631_SM1463.docx]

**Table S1.** CyTOF markers: a 41-dimensional mass cytometry panel

| List | Label | Marker | List | Label | Marker |
| --- | --- | --- | --- | --- | --- |
| 1 | 160Gd | TGF_b | 22 | 152Sm | CD11c |
| 2 | 146Nd | CD66a | 23 | 174Yb | CD31 |
| 3 | 161Dy | CD64 | 24 | 176Yb | CD43 |
| 4 | 173Yb | CD172a | 25 | 151Eu | CD44 |
| 5 | 156Gd | CD66b | 26 | 150Nd | CD14 |
| 6 | 168Er | CD177 | 27 | 163Dy | CD121a |
| 7 | 144Nd | CD16/32 | 28 | 153Eu | PD-L1 |
| 8 | 167Er | CD54 | 29 | 165Ho | Ly-6G |
| 9 | 143Nd | CXCR2 | 30 | 141Pr | MHC-II |
| 10 | 155Gd | CXCR4 | 31 | 169Tm | CD62L |
| 11 | 166Er | CCR2 | 32 | 145Nd | CD24 |
| 12 | 162Dy | CXCR3 | 33 | 159Tb | PD_1 |
| 13 | 154Sm | CD18 | 34 | 175Lu | CD11a |
| 14 | 147Sm | CCR5 | 35 | 171Yb | CD13 |
| 15 | 149Sm | CX3CR1 | 36 | 148Nd | CD36 |
| 16 | 170Er | CD88 | 37 | 157Gd | CD49d |
| 17 | 158Gd | CD10 | 38 | 164Dy | CD16.2 |
| 18 | 89Y | CD45 | 39 | 142Nd | CD127 |
| 19 | 115ln | CD3e | 40 | 172Yb | CD71 |
| 20 | 197Au | CD4 | 41 | 209Bi | CD11b |
| 21 | 198Pt | CD8a |  |  |  |

**Table S2.** Characteristics of patients and healthy people

|  | Sepsis | Health | *P* |
| --- | --- | --- | --- |
| n | 15 | 10 |  |
| Male (%) | 7 (46.67%) | 8 (80.00%) | 0.211 |
| Age (years) | 2.33[1.67, 8.00] | 5.50 [2.96, 9.75] | 0.101 |
| Laboratory data |  |  |  |
| Mean arterial pressure (mm Hg) | 74.67 [63.00, 76.67] | NA | NA |
| White blood cell count (10^9^/L) | 10.92 [7.06, 17.63] | 7.42 [4.89, 7.60] | 0.021^*^ |
| Hemoglobin (g/L) | 121.00 [110, 131] | 130.00 [121.00, 142.00] | 0.146 |
| Hematocrit (%) | 36.00 [32.00, 38.00] | 39.90 [35.90, 42.10] | 0.094 |
| Platelet count (10^9^/L) | 229.00 [177.00, 312.00] | 344.00 [254.00, 370.00] | 0.091 |
| Albumin (g/dL) | 35.20 [33.10, 38.60] | 44.70 [43.93, 45.83] | <0.01^**^ |
| Aspartate aminotransferase (U/L) | 34.00 [21.00, 54.00] | 30.00 [25.50, 33.00] | 0.469 |
| Alanine aminotransferase (U/L) | 27.50 [12.00, 41.00] | 14.00 [11.50, 18.75] | 0.176 |
| Total bilirubin (μmol/L) | 14.65 [5.93, 117.10] | 7.30 [5.73, 10.80] | 0.141 |
| Creatinine (μmol/L) | 27.00 [19.00, 37.00] | 39.00 [28.75, 45.75] | 0.030^*^ |
| INR | 1.18 [0.95, 1.32] | 0.92 [0.86, 0.98] | 0.013^*^ |
| Infection |  | NA | NA |
| gram-positive bacteria (%) | 7 (46.67%) |  |  |
| gram-negative bacteria (%) | 8 (53.33%) |  |  |
| Viral (%) | 0 |  |  |
| Other (%) | 0 |  |  |
| Vasopressors |  | NA | NA |
| No (%) | 14 (93.33%) |  |  |
| Yes (%) | 1 (6.67%) |  |  |
| Unknown (%) | 0 |  |  |
| Mechanical ventilation |  | NA | NA |
| No (%) | 13 (86.67%) |  |  |
| Yes (%) | 2 (13.33%) |  |  |
| Unknown (%) | 0 |  |  |

The data are expressed as the means ± SDs, medians (IQRs) or numbers of patients (percentages). Continuous variables were compared via Student’s *t* test and the Mann‒Whitney *U* test, and categorical variables were compared via the *χ2* test or Fisher’s exact test between patients and healthy people. **P* < 0.05; ***P* < 0.01.
